# Experimental Verification of Objective Visual Fatigue Measurement Based on Accurate Pupil Detection of Infrared Eye Image and Multi-Feature Analysis

**DOI:** 10.3390/s20174814

**Published:** 2020-08-26

**Authors:** Taehyung Kim, Eui Chul Lee

**Affiliations:** 1 Department of Computer Science, Sangmyung University, Seoul 03016, Korea; th_k9304@naver.com; 2 Department of Human-Centered Artificial Intelligence, Sangmyung University, Seoul 03016, Korea

**Keywords:** visual fatigue, infrared eye image, pupil accommodation speed, blink frequency, eye-closed duration

## Abstract

As the use of electronic displays increases rapidly, visual fatigue problems are also increasing. The subjective evaluation methods used for visual fatigue measurement have individual difference problems, while objective methods based on bio-signal measurement have problems regarding motion artifacts. Conventional eye image analysis-based visual fatigue measurement methods do not accurately characterize the complex changes in the appearance of the eye. To solve this problem, in this paper, an objective visual fatigue measurement method based on infrared eye image analysis is proposed. For accurate pupil detection, a convolutional neural network-based semantic segmentation method was used. Three features are calculated based on the pupil detection results: (1) pupil accommodation speed, (2) blink frequency, and (3) eye-closed duration. In order to verify the calculated features, differences in fatigue caused by changes in content color components such as gamma, color temperature, and brightness were compared with a reference video. The pupil detection accuracy was confirmed to be 96.63% based on the mean intersection over union. In addition, it was confirmed that all three features showed significant differences from the reference group; thus, it was verified that the proposed analysis method can be used for the objective measurement of visual fatigue.

## 1. Introduction

Currently, many people spend a significant amount of time viewing electronic displays. With the development of computers and electronic display devices, electronic displays can be seen everywhere. Through continuous development, the electronic display is becoming clearer and more realistic. However, as screen sizes and resolution increase, the amount of information reflected in the eyes, and the movement of the eyes also increases. This contributes to the prevalence of eye fatigue. Digital eye fatigue that accompanies computer vision syndrome is related to the use of computers (desktops, laptops, and tablets) and other electronic displays (smartphones and electronic devices) and is experienced by over 40% of adults and over 80% of teens. Previous studies have shown that preschoolers spend up to 2.4 h a day watching electronic screens, which is an indication of the ubiquity of electronic display devices. Accordingly, eye fatigue problems are increasing and are a central problem in the electronic display field [1].

When eyestrain is low, it can be resolved by simply closing the eyes for a period of time. However, when fatigue accumulates, users may experience pain around the eyes, blurred vision, headaches, dizziness, and other symptoms. The problem is that it is difficult to notice the accumulation of eye fatigue when working with an electronic display. In addition, preschoolers may deliberately ignore fatigue for continued viewing. Therefore, it is important to relieve eye fatigue at the appropriate time, which highlights the need for objective and quantitative criteria for measuring eye fatigue [2]. In order to solve the aforementioned problems, several studies have measured eye fatigue based on images.

In previous research, eye fatigue was measured through a questionnaire [3,4,5]. However, since this questionnaire is based on the subjective thinking of the user, it is difficult to guarantee its reliability. Further studies have used biosignals to measure eye fatigue objectively [6,7,8,9]. Biosignal measurement methods such as galvanic skin response, photoplethysmogram, and skin temperature are objective measurement methods; however, the measurement results may vary due to the surrounding environment, humidity, temperature, and subject movement. In addition, the sensors must be attached to the human body for biosignal measurement, which can cause the subject to feel uncomfortable. In order to solve these problems, some studies have measured eye fatigue based on images. In one such study, eye fatigue was measured based on pupil size values obtained using the device, which is commonly used in ophthalmology [10]. A subsequent study measured eye fatigue using the pupil size of the subject and the time it takes to read an article with an eye-tracker [11]. However, these methods have some key disadvantages; specifically, expensive equipment is required, and the pupil size is influenced by various factors, such as the brightness of the surrounding environment. Yet another study measured the eye fatigue caused by watching an Liquid Crystal Display (LCD) TV by measuring pupil accommodation speed [12]. In this study, the pupil size difference between adjacent frames was used to calculate the pupil accommodation speed. Based on 30 frames, the difference between frames was 33 ms. However, it is difficult to determine whether the difference that occurs in this short period is meaningful. In this study, the points where the pupil size increases and decreases are designated as the inflection points, and the rate of change between the inflection points is then calculated in order to utilize meaningful pupil size differences for analysis. One study aimed at measuring visual fatigue through eye behavior concluded that blinking frequency is an appropriate metric for measuring visual fatigue, but it is impatient to draw conclusions with only one metric [13]. Therefore, in order to confirm an objective and reliable visual fatigue measurement scale, it is necessary to observe various features in various environments. In our study, the pupil area of the subject was extracted and their multi-features of the eye were observed. The multi-features used in our study are defined based on accurate pupil region detection.

To detect pupil region, research that detects the dark and bright pupils has the advantage of being robust against external light changes, but it uses two consecutive frames, making it difficult to detect the pupil if there is violent motion [14]. The method using the circular Hough transform is robust to noise, but detection accuracy is poor when the subject rotates the head or the pupil is not a clean circle [15]. In addition, the circular edge detection method using the ellipse template or the difference in the pixel values of the pupil boundary can accurately detect the pupil region, but the computational complexity is very high because it is applied to the entire image in consideration of the sizes of various circular templates [16]. In the study for gaze tracking in a visible light environment, the pupil region was detected by performing the logical AND operation of two binarized images [17]. This method is capable of real-time detection, but it is highly affected by external light. Finally, the method using binarization, boundary segmentation, and ellipse fitting can obtain the optimal and fast result in high-resolution eye images, but it has a disadvantage in that the detection result varies depending on the location of the device [18]. To solve these problems, a deep learning method is adopted in order to detect pupil region, which can be operated in real-time.

In recent research, studies have been conducted to quantitatively measure eye fatigue through an eye tracker that provides infrared eye images suitable for detecting the pupil region [19,20]. Our study is similar in that it uses infrared eye imaging, and this paper attempts to verify a more suitable quantitative metric for measuring visual fatigue.

Our study has three contributions. First, a method based on noise removal and inflection point detection was used to calculate the exact change in pupil size. Second, a deep learning-based pupil region detection method was used for accurate pupil size calculation. Third, in order to verify the proposed method, experiments were conducted in various environments (gamma conversion, color temperature conversion, and brightness conversion). In addition, the influence of three features (pupil accommodation speed, blink frequency, and eye-closed duration) on the objective and quantitative measurement of eye fatigue was experimentally verified.

The remainder of this paper is organized as follows: In Section 2, the apparatus and methods for measuring eye fatigue, which refers to the overall flow of the experimental method and describes the eye imaging equipment, are presented. In addition, the method of deep learning-based eye region detection is briefly described and the eye fatigue measurement scale used in the experiment is discussed. Section 3 presents the accuracy of the eye region detection and confirms the results of the eye fatigue measurements. This paper is concluded in Section 4 and Section 5 with the final analysis and interpretation of the experimental results.

## 2. Materials and Methods

**Figure 1:**
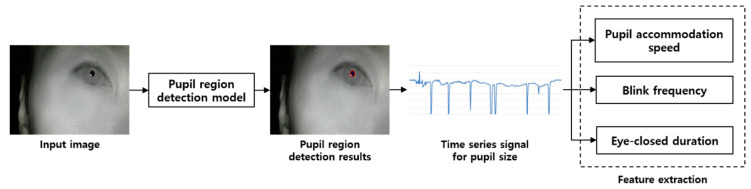
Flow chart of the proposed method for visual fatigue measurement.

This section describes the devices used in the experiment and the pupil detection Algorithm 1. The overall flow of the experiment is shown in Figure 1. First, a head-mounted device equipped with an infrared camera module was worn and an image of the eye, while watching a video to cause visual stimulation, was captured. Next, labeling and size filtering were applied to the results obtained by performing convolutional neural network(CNN)-based semantic segmentation to obtain the pupil size value. Based on the obtained pupil size values, the pupil accommodation speed, blink frequency, and eye-closed duration were calculated, and changes in these three characteristics were analyzed over time.

### 2.1. Equipment Used in the Experiment

**Figure 2:**
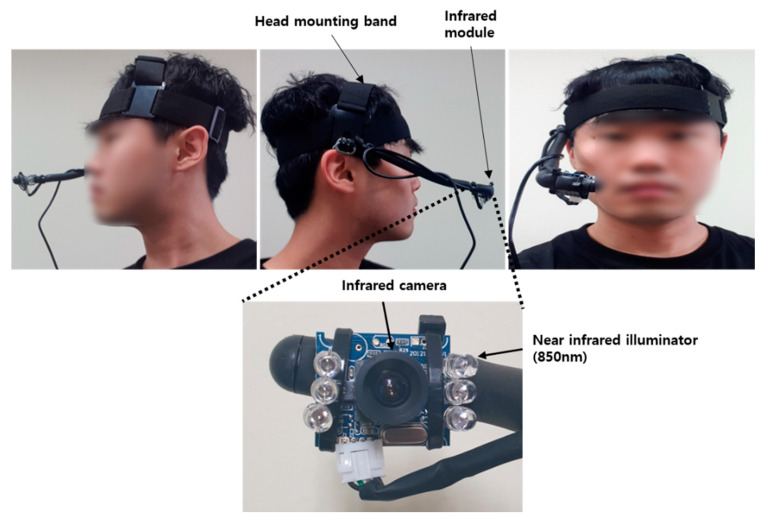
Eye imaging equipment and how it is worn.

**Figure 3:**
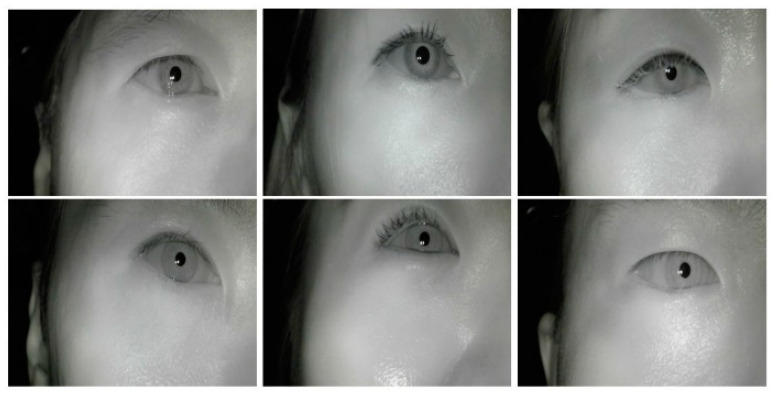
Example of an eye image taken with the near-infrared camera for the experiment.

The equipment for measuring eye fatigue is shown in Figure 2. An infrared camera module was attached to the end of the support connected to the headband. The infrared cameras were connected to a computer via a universal serial bus and video was transmitted at 30 frame per second(fps). In addition, an infra-red(IR) pass-through filter (visible light cutoff) is attached to the camera lens to pass IR light with a wavelength greater than 750 nm, and near-infrared illumination was used to illuminate the pupil area that cannot be seen by the eye with visible light. The wavelength of the used IR illumination is 850 nm, which is invisible to the eye, so it can suppress the dazzling lighting effect. Figure 3 is an example of the input image used in the experiment. Because the subject’s right eye was captured by the head wearable camera, as shown in Figure 2, the image capturing angle is not affected by facial rotation and translation. Next, we describe the method for dividing the eye region from the remaining region in the input image.

### 2.2. Pupil Region Detection Method

**Figure 4:**
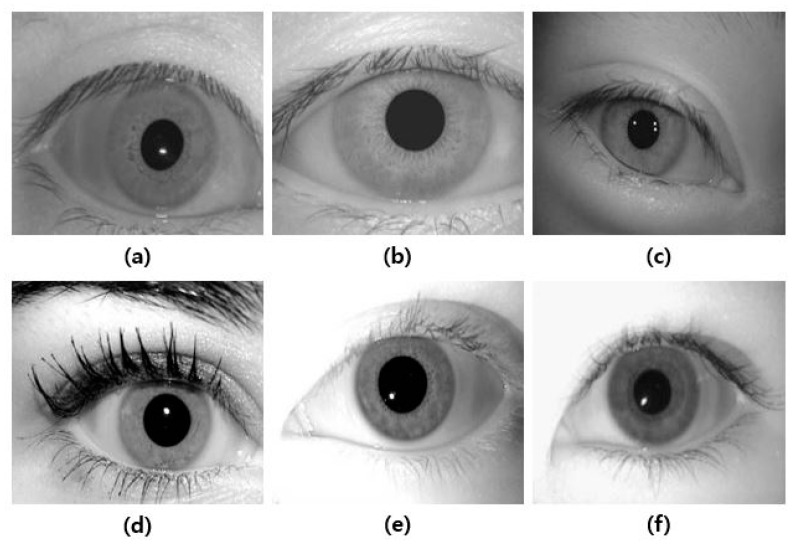
Example of a public database related to eye images: (**a**) Bath database, (**b**) cassia v1, (**c**) cassia v2, (**d**) Iris Challenge Evaluation(ICE)2005, (**e**) ICE 2006, and (**f**) Multimedia university(mmu) database.

Accurate pupil region detection is required to measure the pupil accommodation speed, blink frequency, and eye-closed duration. Several studies have been conducted on detecting pupils using filtering and image processing techniques in facial images, including eyes. The existing methods show good performance in images that are pre-cut to fit the eye area, as shown in Figure 4. However, as shown in Figure 3, if the area occupied by the eye is small and the background area is wide, the detection accuracy is poor. In addition, there is a study to find the pupil center point on the entire face for gaze tracking. However, since an RGB camera is used and minute changes in pupil size cannot be measured, a new pupil region detection method is required [21].

We use the pupil size to extract three features for eye fatigue measurement: the pupil accommodation speed, blink frequency, and eye-closed duration. However, if inaccurate pupil region segmentation occurs frequently, it has a great influence on the feature extraction and analysis steps; thus, it is necessary to accurately detect the pupil region.

**Figure 5:**
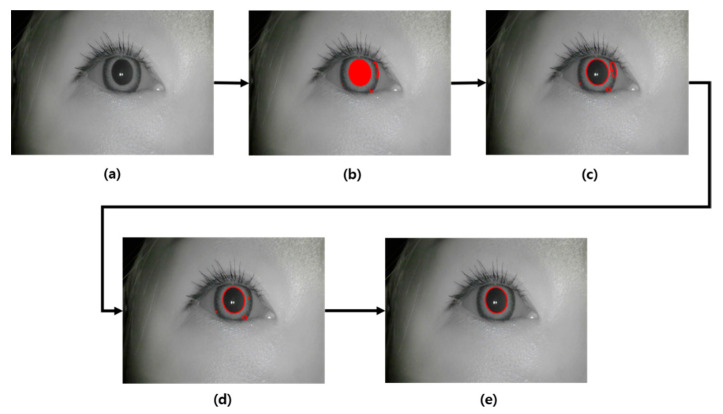
Pupil detection algorithm flow chart: (**a**) Input image, (**b**) results of CNN-based semantic pupil segmentation, (**c**) results of contour detection, (**d**) results of aspect ratio-based filtering, and (**e**) results of size-based filtering and pupil region detection.

The flow chart of our pupil detection algorithm is shown in Figure 5. First, an image is entered into the CNN-based semantic segmentation model to obtain a pupil candidate pixel. The semantic segmentation model uses the DeepLab V3+ model published by Google [22]. Following this, a contour detection algorithm is applied to the pixels predicted to be the pupil region, and adjacent pixels are grouped into candidate groups. In this step, the candidate groups are sometimes generated outside the real pupil region; thus, the incorrectly predicted candidate groups must be removed. Using the fact that the shape of the pupil is almost circular, it is removed as a candidate if the aspect ratio exceeds a preset threshold which is set to 0.4. Second, filtering is performed based on the number of pixels in the remaining candidate groups that do not exceed the threshold. In this study, the threshold of the pupil region’s number of pixels is set to 300 pixels. The thresholds mentioned here were chosen experimentally to obtain the highest Intersection over Union(IoU) results, which are discussed in Section 3.2. As shown in Figure 3, the pupil size in the image does not show a significant difference for each object. Therefore, the candidate groups with unusually large or small numbers of pixels are removed. Finally, if there are no remaining candidate groups, the pupil size of the corresponding frame is determined to be 0; if candidate groups remain, the largest group is determined to be the pupil region.
**Algorithm 1:** Proposed algorithm for pupil detection in a video frame.1: **Inputs:** Video frame F2: **Output:** Contour size fitted to the pupil area3: **Begin**4:  CA_THR = 3005:  AS_THR = 0.46:  Input F to CNN-based semantic segmentation model and output SF (Segmented F)7:  Contours detection in SF8:  **for** contour **in** contours:9:     Calculate area value CA from contour10:      **if** CA > CA_THR:11:        Calculate width (W) and height (H) from contour12:            **if** W / H > AS_THR:13:         C_EF output by performing ellipse fitting for contour14:  **End**15:    Area calculation of C_EF16: **End**


**Figure 6:**
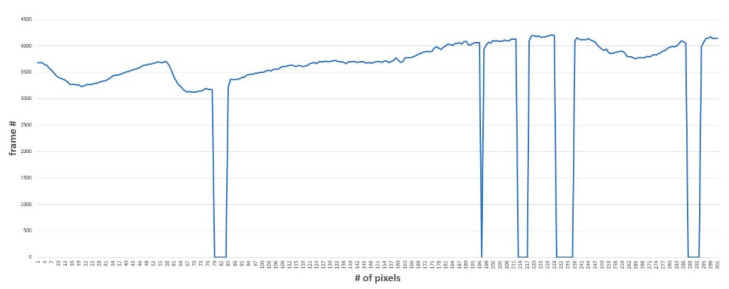
Time series signal example for pupil size: x-axis: pupil size value (unit: pixel); y-axis: frame number.

After applying the algorithm in Figure 5, ellipse fitting is performed on the detected pupil area and the size of the ellipse is calculated and used for feature extraction. The graph representing the calculated area value in frame units over time can be seen in Figure 6. The graph in Figure 6 is the resulting graph for the eye image taken for 10 s at 30 fps, the x-axis is the frame, and the y-axis is the pupil size. If the frame does not have a pupil, the pupil size value is treated as 0. 

### 2.3. Eye Fatigue Measurement Scale

Next, we calculated the pupil accommodation speed from the pupil size data. In our previously published paper, the pupil accommodation speed was calculated using the size difference between adjacent frames [12]. However, since pupil convergence and divergence proceed over several seconds, the pupil accommodation speed calculated using this size difference is not appropriate. In addition, using a static interval difference may produce different results depending on changes in the surrounding environment. Thus, to calculate the accurate pupil accommodation speed, we designated the point at which the pupil contraction and expansion were switched as the inflection point and calculated the pupil accommodation speed through this point.

**Figure 7:**
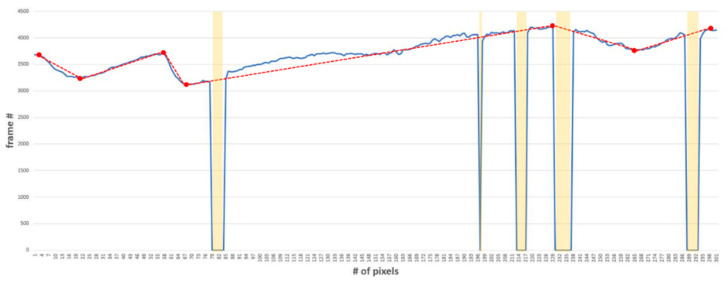
Example of pupil size inflection point detection considering eye blinking. Red points: detected inflection point, red dotted line: express pupil accommodation speed, yellow bars: blink point and eye-closed duration.

The inflection points and the point where the pupil size is zero (from the graph in Figure 6 are displayed in Figure 7. Changes in the size values continue to occur, but not all are significant. Therefore, it is necessary to find an appropriate inflection point, such as the point indicated by the red dot in Figure 7. In order to determine an appropriate inflection point, all inflection points present in the data are first found—these are called inflection point candidates. The inflection point candidates are selected as inflection points if two conditions are satisfied: (1) When the difference value sign from the previous inflection point is different from the difference value sign first calculated, and (2) when the difference value from the previous inflection point exceeds the threshold.

Three features are used to measure visual fatigue:

1. Average pupil accommodation speed
2. Blink frequency per unit time
3. Average time to eye-closed duration

The pupil accommodation speed was calculated using Equation (1), where *N* is the total number of inflection points minus one. In addition, *S_n_* and *S*_*n*+1_ represent the size values of the *n*th and (*n* + 1)th inflection points, and *F_n_* and *F*_*n*+1_ represent the frame numbers of the *n*th and (*n +* 1)th inflection points. Blink frequency is calculated as the number of times the pupil size value shown by the yellow bar in Figure 7 is zero. In this case, the number of times the pupil size value was zero is five, and the eye blinked 0.5 times per second in 10 s. The eye-closed duration is calculated using the number of frames in which the size value is zero. These frames were marked as yellow bars in Figure 7. For example, because the numbers of frames at the point where the size value is zero were 6, 1, 5, 8, and 6, the eyes were closed for 26 frames within 10 s. Changing the unit from frames to seconds results in 0.86 s, and the average closing time for the duration (10 s) is 0.086 s.

(1) $$P=\;\frac{1}{N}\overset{N}{\underset{n=1}{\sum }}\left|\frac{S_{n+1}-S_{n}}{F_{n+1}-F_{n}}\right|$$

The extracted features were selected based on three hypotheses: First, the pupil accommodation speed decreases as the iris control muscles feel tired over time. Second, the blink frequency increases fatigue and dryness over time. Third, if tiredness or dryness is felt, the eyes are closed for a longer period of time.

## 3. Results

### 3.1. Experimental Setup

**Figure 8:**
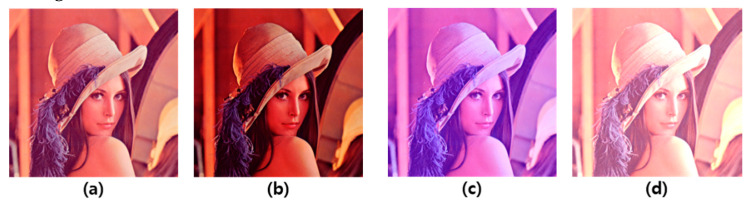
Examples of optical changes applied to images for experiments: (**a**) Original, (**b**) gamma conversion, (**c**) color temperature conversion, and (**d**) brightness conversion.

The experiment was conducted on 20 subjects watching a tablet in a room that blocked all ambient light to cause visual fatigue. In order to cause visual fatigue, the subjects watched a 16-min video of scenes with frequent changes in brightness and content. In addition, diversity of the viewing environment was added through changes in gamma, color temperature, and brightness of the display. An example of optical changes in images for various visual environments can be seen in Figure 8. Because of copyright issues, these were expressed as Lena images instead of actual images. In the case of gamma conversion, a gamma value of 2.0 was added to sharpen the contrast; in the case of color temperature changes, the blue value was increased to induce more blue light. Lastly, in the case of brightness change, the brightness of the entire image was increased.

### 3.2. Pupil Region Detection Results

**Figure 9:**
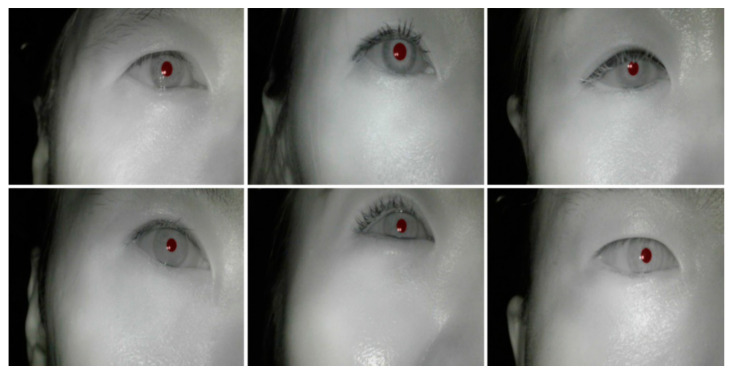
Semantic segmentation model results. The extraction results are marked in translucent red.

The training and performance evaluation of the segmentation model were performed using eight data points from all the data. The first minute in a video was used as a reference frame and the remaining 15 min were used to measure fatigue. In addition, 1800 frames were extracted from the reference frame at a rate of 3 fps, and a total of 4800 frames were used as the dataset for training. From this dataset, 3360 frames were divided into training sets and 1440 frames were divided into test sets for model performance evaluation. As shown in Figure 3, the position and shape of the pupil region in the image look similar for each person. Therefore, the generalization performance was excluded from consideration, and the dataset was constructed to achieve high accuracy. Both training and testing were performed in the NVIDIA GeForce GTX 1080 Ti (Santa Clara, CA, USA) environment. The processing speed was 20 fps for 640 × 480 images and 30 fps for 320 × 240 images. For this experiment, 640 × 480 images were used for precise region detection. The prediction results are shown in Figure 9.

The accuracy of pupil detection was confirmed by the segmentation model accuracy: pixel IoU was used as a model performance evaluation scale. The model performance was verified by calculating the mean IoU (mIoU), which is the average of the accuracy results for the entire test set. Thus, the mIOU was confirmed to be 96.63. In addition, performance evaluation was conducted using the CASIA-Iris V3 database. A total of 2639 images were used in the test, and the mIOU value was found to be 97.54. From these results, it is evident that the detection accuracy was improved through post-processing and the performance did not affect the fatigue analysis results.

### 3.3. Eye Fatigue Measurement Results

**Figure 10:**
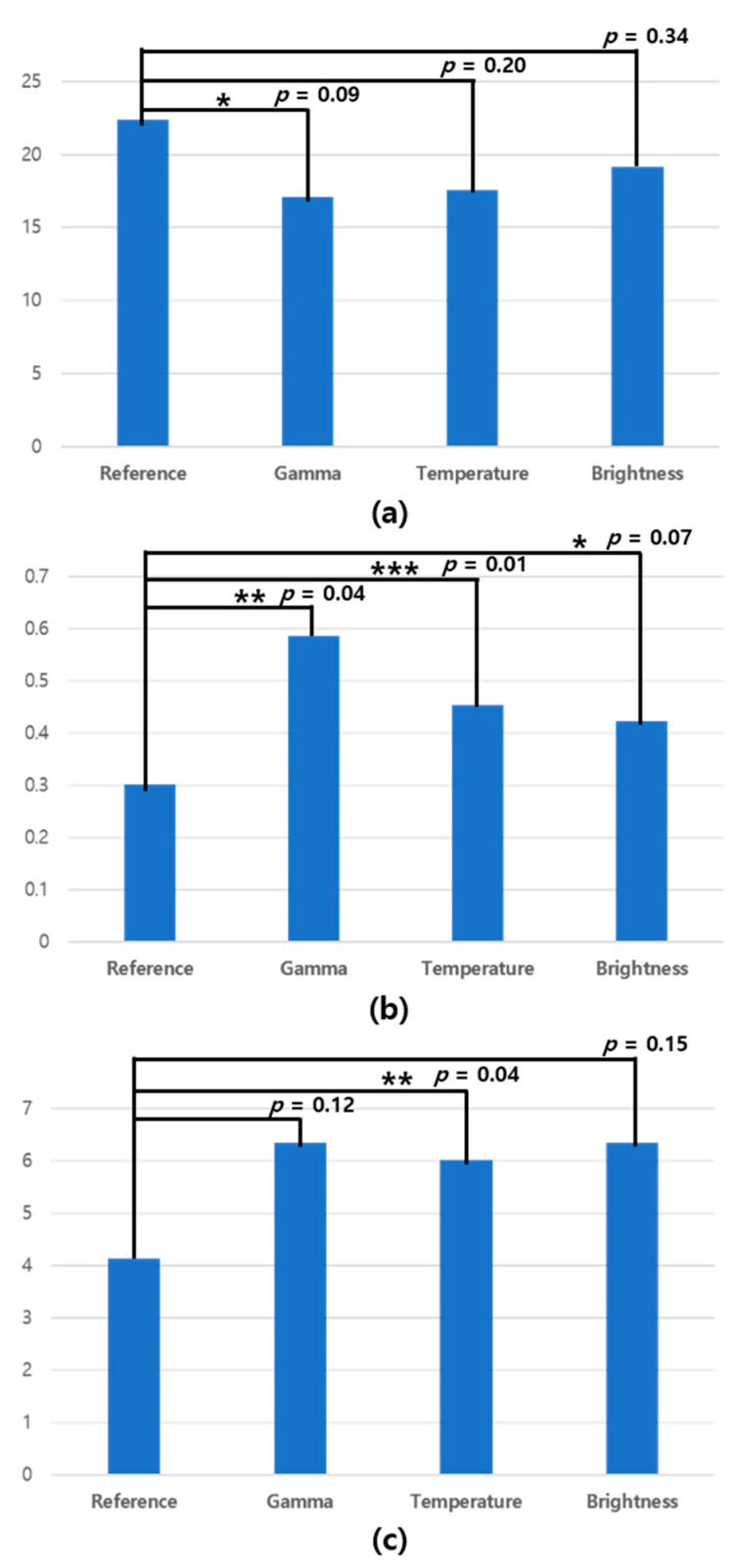
Statistical significance comparison results of three eye features: (**a**) Pupil accommodation speed, (**b**) blink frequency, and (**c**) eye-closed duration (***, **, and * mean statistically significant at a confidence level of *p* < 0.01, 0.05, and 0.1, respectively).

The results of measuring visual fatigue while watching on the tablet can be seen in Figure 10, which displays the optical changes (original, gamma, color, temperature, and brightness) applied to the image in order from the left of each graph. Additionally, detailed values can be seen in Table 1.

The statistical significance between the viewing environment with no optical change and the viewing environment with optical change was confirmed, and this value is expressed as a *p*-value. In Figure 10, the y-axis of (a) is the number of pixels, which represents the degree to which the number of pixels corresponds to the eye area changes per second. The y-axis of (b) is the number of frames, which represents the number of times the eye blinks per second. In addition, the y-axis of (c) is the number of frames, which means the number of frames with the eyes closed for one second.

The interpretation of the experimental results in Figure 10 is as follows. In (a), the pupil accommodation speed for reference stimulus was greater than the results of the other stimuli. This result can be interpreted that the reference stimulus causes less visual fatigue than other stimuli. In (b), the blink frequency for the reference stimulus was observed to be lower than the cases for other stimuli. Assuming that frequent blinking is a metric for high visual fatigue, this result can be regarded that the reference stimulus caused the least visual fatigue. As shown in (c), the eye-closed duration for the reference stimulus was lower than that of other stimuli. In a similar interpretation to (b), this result supports the finding that the reference stimulus causes the least visual fatigue. As a result, all three features, such as pupil accommodation speed, blink frequency, and eye-closed duration, were found to support the hypothesis that modified visual stimuli cause higher visual fatigue than reference stimulus. In addition, from the results (a)~(c), it was confirmed that the modification of gamma induces visual fatigue higher than temperature and brightness in common.

## 4. Discussion

The results of our experiments confirmed that the pupil accommodation speed was lowered in all environments. We also confirmed that our hypotheses for all three features in the tablet viewing environment matched. As shown in Figure 10b, the *p*-value for the blink frequency was less than 0.05 in the gamma and color temperature change environments. In addition, Figure 10c shows a significant difference, as the *p*-value for the eye-closed duration in the color temperature change environment was less than 0.05.

Previous studies have shown that visual fatigue increases under different environmental conditions [12]. Based on the results of this study, significant changes in each change environment (gamma, color temperature, and brightness) were identified. In previous research, which confirmed the degree to which the resolution of the visual display terminal (VDT) affects visual fatigue, the pupil accommodation speed was used as a visual fatigue evaluation scale and a significant difference between the two resolutions was confirmed [23,24]. In the experimental environments of this study, the blink frequency increased with time. In previous research, the visual fatigue of motorists was measured by calculating the eye-closed duration. However, this research did not determine whether the eye-closed duration is suitable for measuring visual fatigue. To solve this problem, our study experimentally verified that the eye-closed duration is suitable for measuring visual fatigue. It has also been argued that visual fatigue occurs when the pupil size changes continuously due to uneven stimulation [24]. In addition, studies have shown that pupil size is an appropriate metric for visual fatigue [25,26]. In fact, one study showed that the pupil accommodation speed is lowered when feeling visual fatigue [12]. Based on previous studies and our experimental results, we can conclude that the pupil accommodation speed, the blink frequency, and the eye-closed duration are all appropriate metrics for visual fatigue.

## 5. Conclusions

In this study, we investigated the pupil features suitable for visual fatigue measurement. While viewing a tablet, eye images were obtained using an infrared camera attached to an HMD, and optical changes were applied to the video to cause visual fatigue. Using the CNN-based semantic segmentation model, the pupil region was extracted from the eye image. Three features extracted from the extracted pupil region were analyzed to measure visual fatigue. The results of this analysis showed significant differences in all three features. That is, visual fatigue causes a decrease in the ability of the iris muscle to move, which slows down pupil size control. In addition, the dry corneal surface due to increased visual fatigue causes frequent blinking. Based on the results of this study, it is expected that visual fatigue can be measured in flight stability improvement systems [27], Virtual reality equipment, video conference systems, the video content production field, and the post-traumatic stress disorder treatment. In addition, this result can be applied to various studies (emotion recognition, gaze tracking, etc.) using infrared cameras. However, the 16-minute viewing time is a short time to cause visual fatigue. Therefore, further studies will increase viewing time to cause enough visual fatigue and improve the method of extracting pupil characteristics for real-time measurements.

## Figures and Tables

**Table 1:** Three eye feature extraction results according to display device and color component adjustments.

Eye Feature	Color Component Adjustment			
	Reference	Gamma	Temperature	Brightness
Pupil accommodation speed (frame/s)	22.4088	17.1234	17.5438	19.1254
Blink frequency (blink/s)	0.3014	0.5876	0.4531	0.4222
Eye-closed duration (frame/s)	4.1254	6.3458	6.0125	6.3485
